# The Effect of Kyphoplasty on Opioid Use in Patients With Vertebral Compression Fractures

**DOI:** 10.7759/cureus.54084

**Published:** 2024-02-12

**Authors:** Ben Silverman, Frances Shofer, Kirk Bonner, Stephen Hampton

**Affiliations:** 1 Physical Medicine and Rehabilitation, Hospital of the University of Pennsylvania, Philadelphia, USA; 2 Emergency Medicine, University of Pennsylvania Perelman School of Medicine, Philadelphia, USA

**Keywords:** vertebral compression fracture, osteoporosis, opioid use, vertebral augmentation, kyphoplasty

## Abstract

Objective

The primary objective of this study was to assess opioid use in the 90 days following kyphoplasty (KP) compared to the period between compression fracture and KP.

Methods

All patients aged 50-85 who underwent KP following a newly diagnosed vertebral compression fracture (VCF) at a large, urban academic medical center between January 1st, 2015, and January 1st, 2023, were screened for inclusion. Patients were excluded if they had an opioid prescription in the month prior to the compression fracture, had a history of malignancy, or underwent concomitant or other surgical procedures in the 90 days following KP. Opioid measures, including the prescribed amount of morphine milliequivalents (MME) per day, number of opioid days, and total MME (MME per day x number of opioid days), in addition to numerical rating scale (NRS) pain scores, were analyzed pre- and post-KP.

Results

A total of 27 patients met the eligibility criteria, with a mean age of 69.7 and 59.2% being female. Sixteen patients (59%) had received an opioid prescription between compression fracture and KP (opioid group). The median differences pre- and post-KP in prescribed MMEs per day, number of opioid days, and total MMEs were 17.7 (p=.0009), 11.0 (p=.0004), and 232.5 (p<.0001), respectively. There was a significant difference in NRS pain scores in both the opioid group (6.25, p<.0001) and the non-opioid group (4.36, p<.0001) pre- and post-KP.

Conclusion

Our findings suggest that KP may be associated with a reduction in both opioid use and pain scores in opioid-naïve patients with VCFs. Larger studies that directly compare KP to conservative management are needed to fully assess the impact of KP on opioid and pain outcome measures.

## Introduction

There are approximately 1.5 million vertebral compression fractures (VCFs) annually in the general US population [1]. The prevalence of this condition increases with age and has been shown to reach 40% by age 80 [2]. Women tend to be more affected, with an incidence of 10.7 VCFs per 1000 compared to 5.7 per 1000 men [3]. The most common precipitating factor for VCFs is osteoporosis, a progressive bone loss condition affecting 50% of people over 50 years of age [4]. As bones become weak and brittle, it is not uncommon for fractures to develop secondary to low-impact trauma or even normal activity [5]. VCFs constitute 25% of osteoporotic fractures, often at the midthoracic and thoracolumbar junctions [6]. Less common etiologies of VCFs include trauma, malignancy, and infection [1]. VCFs have the potential to cause significant pain, functional limitations, and severe disability, in addition to placing a massive burden on healthcare resources [7].

Initial treatment approaches include conservative management (CM) consisting of analgesia, bracing, physical therapy, and even periods of bed rest [8]. Elderly patients tend to not tolerate bracing, citing joint problems, pain, and difficulty donning and doffing the brace [9]. Thus, elderly patients tend to require longer periods of bed rest, which predisposes them to additional medical complications, including pressure ulcers, venous thromboses, pulmonary complications, and progressive deconditioning [10]. Each additional day of immobility leads to decreased functional capacity, with notable losses in muscle strength and bone mineral density as high as 15% and 1% per week, respectively [11]. To counter acute debilitating pain episodes and allow for the management of ongoing pain, patients are often prescribed pain regimens that include opioids [12]. Opioids are known to cause numerous adverse effects, including decreased gastrointestinal motility and respiratory function, physical dependence, and cognitive impairments [13]. Even low-dose opioid use has been shown to be associated with negative outcomes in elderly populations and may be associated with as many functional problems as the VCF itself [14-15].

Patients with refractory or persistent pain following CM may be candidates for minimally invasive vertebral augmentation (VA) procedures. Kyphoplasty (KP) is an image-guided VA procedure that utilizes balloon inflation into the fracture site prior to cement injection with the goal of providing restoration of vertebral height, stabilization, and pain relief [16]. There has been mixed evidence in the literature regarding the effectiveness of VA and the benefit it may have when compared to CM [17]. The mixed and inconclusive evidence has been mostly due to the poor study design and quality of the studies performed [18]. Several studies have shown a mortality benefit for patients undergoing VA for refractory pain following VCF when compared to CM [19-20]. On the contrary, a large retrospective cohort analysis found no significant difference in mortality between VA and CM when accounting for selection bias [21]. To our knowledge, there has been only one study that has looked at opioid patterns as the primary outcome following VA; however, this included both vertebroplasty (VP) and KP [22].

The primary objective of this study was to assess opioid use in the 90 days following KP compared to the period between compression fracture and KP.

## Materials and methods

Study design 

This was a retrospective cohort study that assessed opioid use and pain scores in patients pre- and post-KP following newly diagnosed VCFs and was conducted at the Hospital of the University of Pennsylvania, which is located in Philadelphia. This study was deemed exempt from review by the University of Pennsylvania Institutional Review Board (Philadelphia, Pennsylvania), and informed consent was waived because the dataset was deemed deidentified.

Participants 

This study identified patients aged 50 to 85 who underwent KP following newly diagnosed VCFs between January 1st, 2015, and January 1st, 2023, at a large, urban academic medical center. This age range was selected to assist in obtaining a population that was inclusive of osteoporotic VCFs. KP procedures were identified using the Current Procedural Terminology procedural code of 62323.

Patients were excluded from analysis if they were not opioid naïve, had a history of malignancy, or underwent concurrent or any surgical procedure in the 90 days following KP. Opioid naïve was defined as not having any opioid prescription in the month prior to the diagnosis of VCF. This criterion was selected to exclude patients with prior or future opioid exposure that was unrelated to the VCF and KP. 

Outcomes

The primary outcome was opioid use in the 90 days following KP compared to the period between compression fracture and KP. During each period, opioid use was approximated by using the prescribed amount of morphine milligram equivalent (MME) per day, number of opioid days, and total MME (MME per day x number of opioid days). Opioid prescriptions were searched using an independent statewide electronic prescription drug monitoring program. This included multiple states that were in close proximity to the academic medical center. Secondary outcomes included pre- and post-KP pain scores. Pain scores were recorded on the numerical rating scale (NRS) at the initial preoperative visit and the first postoperative visit.

Statistical analysis 

Summary statistics are presented as frequencies and percentages for categorical demographic variables, means with standard deviation (SD) for age and pain scores, and median with interquartile range (IQR) for opioid use and days to KP following diagnosis of VCF. To determine differences pre/post-KP for changes in pain intensity, a two-way analysis of variance in repeated measures was performed, where group (opioid prescription between compression fracture and KP) was a fixed factor and time (pre/post) was the repeated measure. Within the group with an opioid prescription prior to KP, changes in opioid use pre/post-KP were determined using the Wilcoxon signed rank test. All analyses were performed using SAS statistical software (version 9.4, SAS Institute, Cary, NC). Figures were created using GraphPad Prism (version 9.3.1, GraphPad Software, San Diego, CA, USA).

## Results

**Figure 1:**
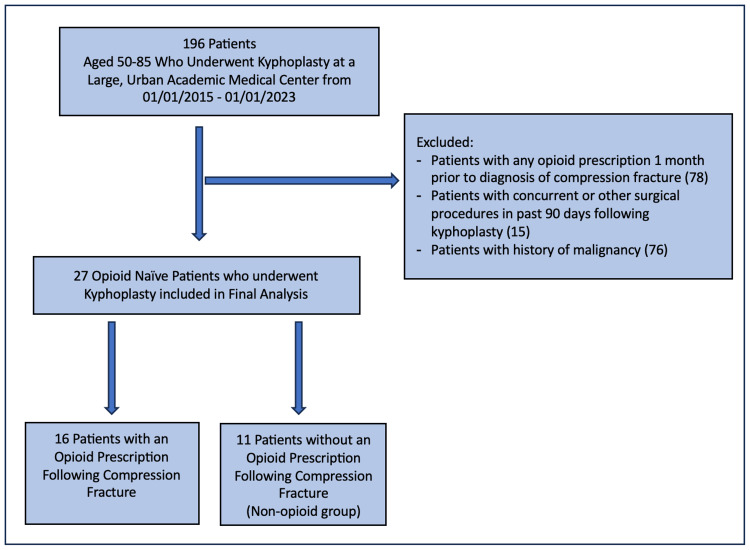
Flow diagram of inclusion and exclusion criteria in the construction of the final cohort

Of the 196 patients identified as having undergone KP, 27 (14%) met inclusion/exclusion criteria and were included in the analysis (Figure 1). Of these 27 patients, 16 (59%) received an opioid prescription following the diagnosis of a compression fracture. The overall mean ± SD age of the study cohort was 69.7 ± 11.04, with the majority female (59.2%) and white (77.8%). Medicare was the most common listed insurance (55.6%), followed by commercial insurance (33.3%) and Medicaid (11.1%). Of the population, 51.9% had a fracture between the levels of L1-L4, 37% between T8-T12, and 11.1% between T4-T7. Around 22.2% of patients underwent a multilevel procedure. The median time to KP was 70 days (IQR: 42-138) (Table 1).

**Table 1:** Demographic characteristics of patients receiving kyphoplasty SD: standard deviation; IQR: interquartile range

Variable	Category	n (%)
Age in years (mean ± SD)		69.3 ± 11.0
Sex	Female	16 (59.2)
	Male	11 (40.8)
Race	White	21 (77.8)
	Black	3 (11.1)
	Other	3 (11.1)
Opioid prescription prior to kyphoplasty	Yes	16 (59.3)
	No	11 (40.7)
Insurance	Medicare	15 (55.6)
	Commercial	9 (33.3)
	Medicaid	3 (11.1)
Vertebral level	T4-T7	3 (11.1)
	T8-T12	10 (37.0)
	L1-L4	14 (51.9)
Multilevel procedure		6 (22.2)
Days to kyphoplasty (median & IQR)		70 (42-138)

Opioid use 

**Figure 2:**
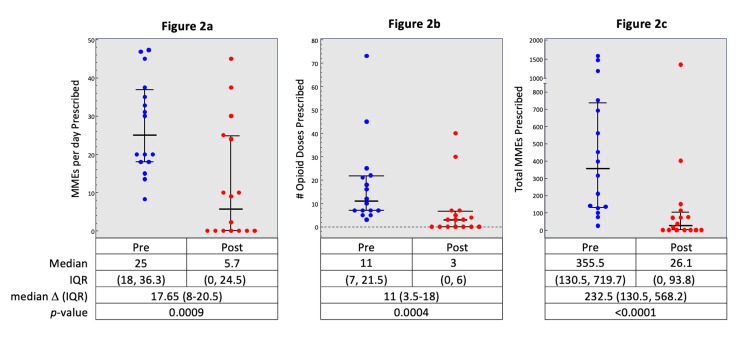
Differences pre- and post-KP in opioid usage including MMEs per day prescribed (2a), opioid days prescribed (2b), and total MMEs prescribed (2c) MME: morphine milligram equivalent; IQR: interquartile range

Of the 27 patients, 16 (59%) had an opioid prescription following VCF. Each of the three opioid measures significantly decreased between pre- and post-KP. Median MMEs per day decreased from 25.0 to 5.7 with a median difference of 17.7 (IQR: 8.0-20.5, p=.0009, Figure 2a). Similarly, median opioid days prescribed also decreased (11 vs. 3 days, median difference: 11.0, IQR: 3.5-18.0, p=.0004, Figure 2b). Lastly, total MMEs decreased from 355.5 to 26.1 (median difference: 232.5, IQR: 130.5-568.2, p<.0001, Figure 2c). 

Pain scores

**Figure 3:**
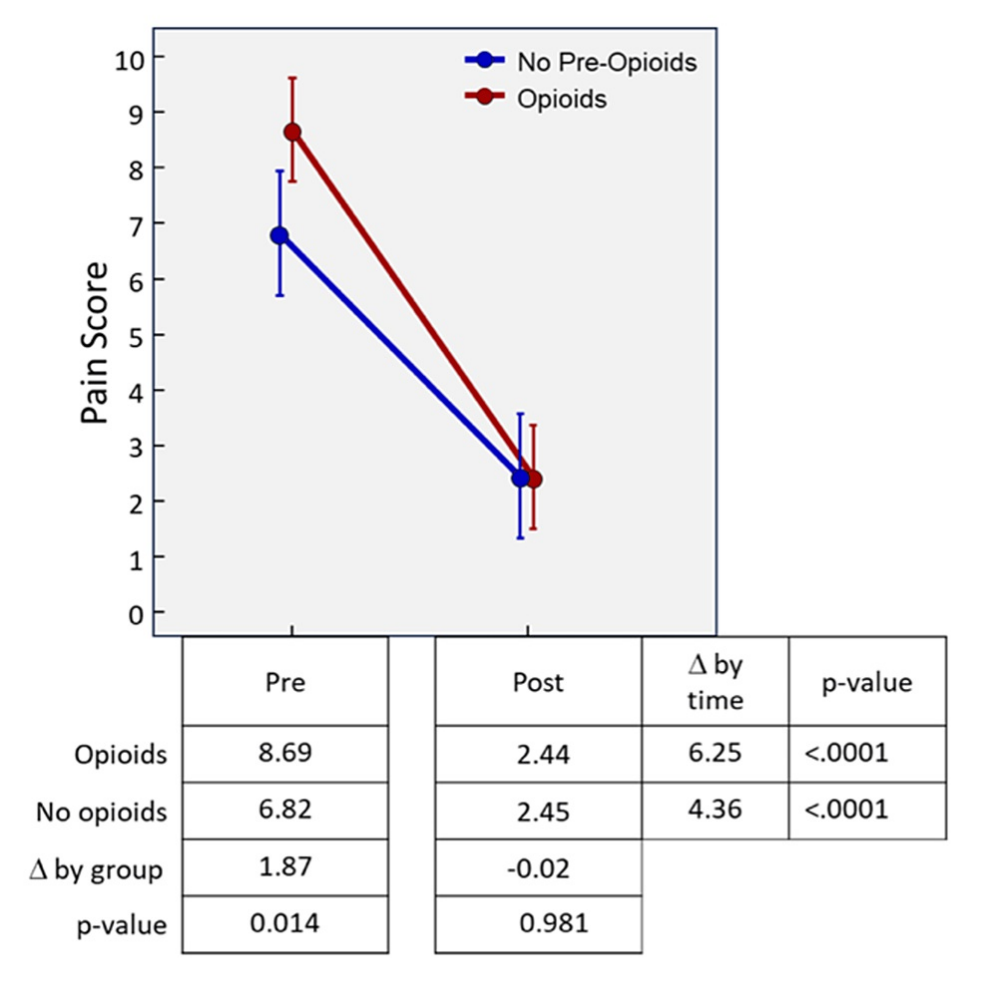
Numerical rating scale pain scores pre- and post-KP

Mean pre-KP pain scores differed by opioid group, where those with an opioid prescription had higher pain scores compared to the non-opioid group (8.69 vs. 6.28, difference=1.87, p=.014, Figure 3). Both groups had a significant reduction in pain score post-KP (difference=6.25 and 4.36 for both opioid and non-opioid groups, respectively, p<.0001). There was no significant mean difference in post-KP pain scores between groups (2.44 vs. 2.45 for opioid and non-opioid groups, respectively, with a difference of -0.02, p=.981). 

## Discussion

This retrospective analysis primarily evaluated the effect of KP on opioid utilization as measured by prescribed MMEs per day, number of opioid days, and total MME. Trends in opioid use were evaluated in the 90 days following KP relative to the use in the period between compression fracture and KP. Overall, the median change in opioid use was 17.65, 11, and 232.5 for prescribed MMEs per day, number of opioid days, and total MMEs, respectively.

Although no study specifically evaluates opioid patterns as a primary outcome following KP alone, our observations were consistent with prior evidence that opioid use decreases following VA. A recently published large retrospective analysis found that 57.1% of VCF patients who underwent VA, including KP or VP, either discontinued or had reduced prescription refills within seven months after the procedure when compared to preoperative baseline levels [22]. A randomized controlled trial that compared KP to conservative treatment measures found a statistically significant reduction in opioid use in the KP group after six months, although this difference was not observed at the 12- or 24-month mark [23]. Furthermore, a multi-center trial that compared KP to VP found that the percentage of patients treated with opioids was significantly reduced at the six-month follow-up mark relative to baseline in both cohorts [24].

A secondary outcome of this study was the assessment of pain scores on the NRS at the initial preoperative visit and the first postoperative visit. A statistically significant reduction of 6.25 and 4.36 were found over this period in the opioid and non-opioid groups, respectively. Our findings are consistent with prior studies that have demonstrated a decrease in pain scores following KP [25-26]. One of the most prominent studies was a large multi-center prospective clinical study that demonstrated a statistically significant reduction in NRS for up to one year in patients with osteoporotic VCFs who underwent KP [27]. This study had a similar patient population to the present study, including an average age of around 70, a majority of women, and 98% of the VCFs caused by osteoporosis.

One notable observation of our study was that the opioid group appeared to be in more pain in the preoperative period compared to the non-opioid group, as detailed by an NRS of 8.69 compared to 6.82. These severe initial NRS pain scores in the opioid group likely explain the need for opioids in the period between compression fracture and KP.

Opioids can be an effective way to manage pain episodes but come at the expense of numerous adverse effects, which even at low doses can affect the elderly population [15]. One retrospective study found opioid use longer than 12 weeks in geriatric patients is associated with an 80% increase in opioid-associated adverse reactions compared to short duration of use [28]. This highlights the importance of reducing and ultimately discontinuing opioid regimens as much as possible in this patient population. Opioid tracking is especially important in patients with osteoporotic VCFs, as the average age tends to be between 70 and 80 years.

This study was clinically meaningful as it added to the paucity of literature surrounding the effect of KP on opioid use.

Study limitations 

This study has several limitations due to its nature as a retrospective analysis. The most notable limitation was the inability to track true opioid consumption, as we are unable to say for certain the number of pills taken from a patient's prescription. Additionally, opioid data could have been influenced by prescriber variability rather than patients' needs. To address this limitation, we included multiple opioid tracking measures and concomitant pain scores to give a more complete picture for each patient. A second limitation was the small size of our study population. This was mostly due to obtaining a true opioid-naive population, which required certain exclusion criteria to be applied.

Furthermore, there was no standard date for preoperative and postoperative visits. These typically ranged between one and two weeks before and after the procedures. The timing of these visits may have an impact on the NRS pain score. Lastly, this study had six patients who underwent a multilevel KP, which may exacerbate pain scores and opioid use.

## Conclusions

Our findings suggest that KP may be associated with a reduction in opioid use and pain scores in opioid-naïve patients with VCFs. KP may be a good alternative to CM; however, larger studies directly comparing KP to CM are needed to fully assess the impact of KP on opioid and pain outcome measures.
